# Supplementary material for: Application of ATAC-Seq for genome-wide analysis of the chromatin state at single myofiber resolution
Source: eLife. 2022 Feb 21;11:e72792. doi: 10.7554/eLife.72792 (PMC8901173; doi:10.7554/eLife.72792)
Supplement: Source data 1. — (A) Unlabeled agarose gel (1.25%) of MuSC ATAC-Seq sequence ready libraries. (B) Unlabeled agarose gel (1.25%) of uninjured myofiber ATAC-Seq sequence ready library. (C) Labeled agarose gel (1.25%) image of MuSC and uninjured myofiber ATAC-Seq sequence ready libraries. (D) Raw file of bioanalyzer results from single myofiber sequence ready ATAC-Seq libraries. [file elife-72792-data1.zip › Figure 1 figure supplement 1 source data 2.pdf]

Assay Class: High Sensitivity DNA Assay
Data Path: C:\...-08-04\BAN300_High Sensitivity DNA Assay_2020-08-04_001.xad

Created: 8/4/2020 11:44:53 AM
Modified: 8/4/2020 12:26:12 PM

Electrophoresis File Run Summary

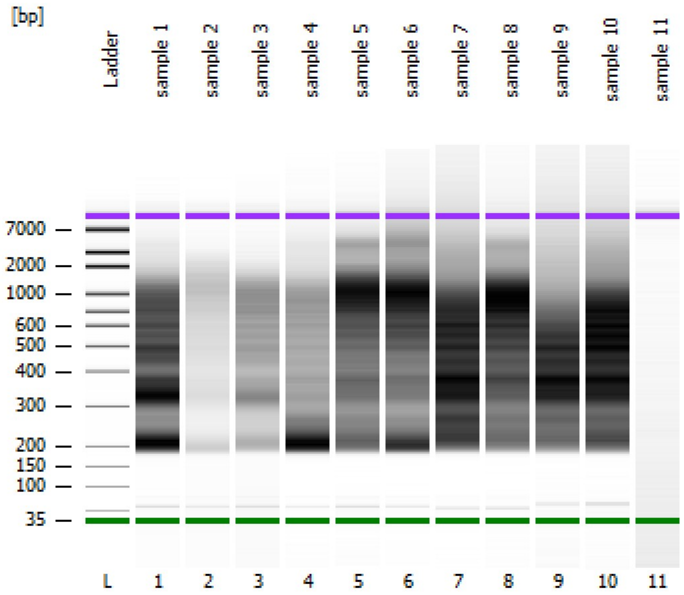

Instrument Information:

Instrument Name: BioA
Firmware: C.01.069
Serial#: DE72900930
Type: G2939A

Assay Information:

Assay Origin Path: C:\Program Files (x86)\Agilent\2100 bioanalyzer\2100 expert\assays\dsDNA\High Sensitivity DNA.xsy

Assay Class: High Sensitivity DNA Assay

Version: 1.0

Assay Comments: Copyright © 2003-2009 Agilent Technologies

Chip Information:

Chip Lot #:

Reagent Kit Lot #:

Chip Comments:

sample 1

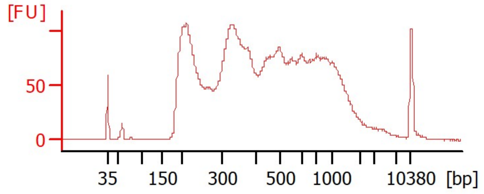

sample 2

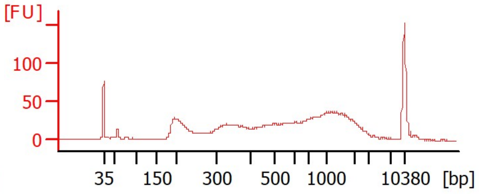

sample 3

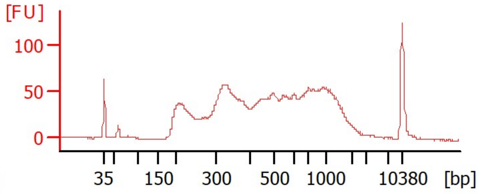

sample 4

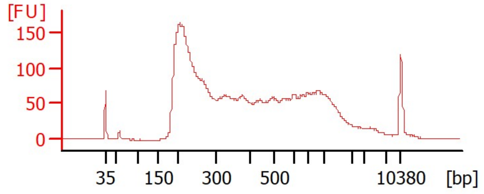

sample 5

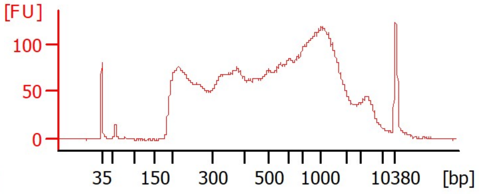

sample 6

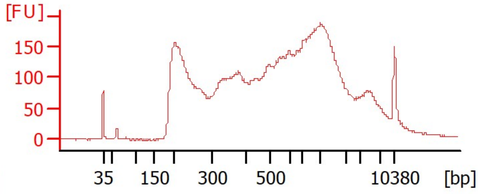

sample 7

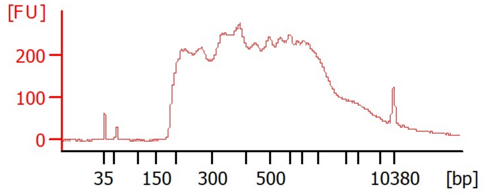

sample 8

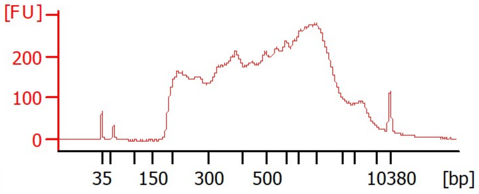

sample 9

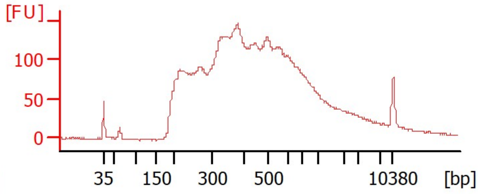

sample 10

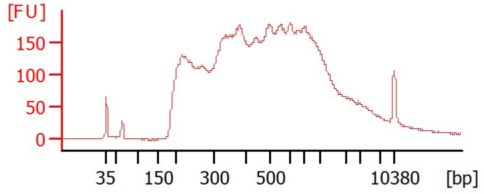

sample 11

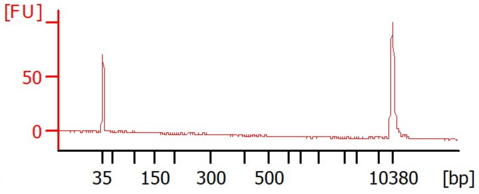

Ladder

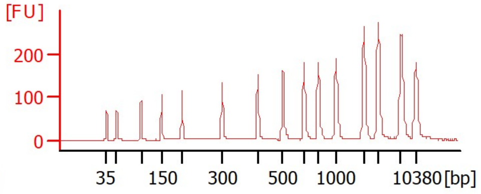

© Copyright 2003 - 2009 Agilent Technologies, Inc.
