# Supplementary figures and images for: Application of ATAC-Seq for genome-wide analysis of the chromatin state at single myofiber resolution
Source: eLife. 2022 Feb 21;11:e72792. doi: 10.7554/eLife.72792 (PMC8901173; doi:10.7554/eLife.72792)

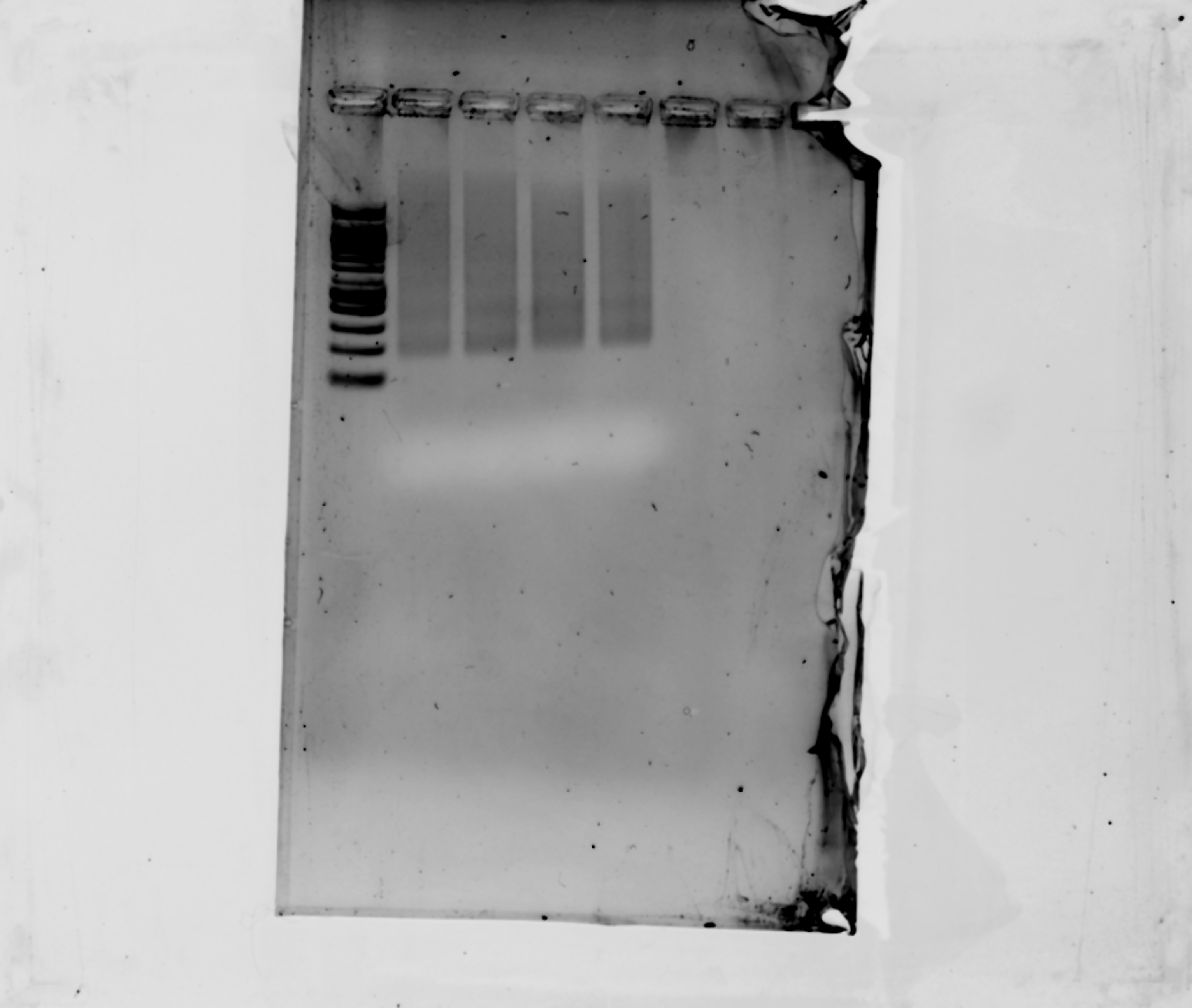

Supplement: Source data 1. — (A) Unlabeled agarose gel (1.25%) of MuSC ATAC-Seq sequence ready libraries. (B) Unlabeled agarose gel (1.25%) of uninjured myofiber ATAC-Seq sequence ready library. (C) Labeled agarose gel (1.25%) image of MuSC and uninjured myofiber ATAC-Seq sequence ready libraries. (D) Raw file of bioanalyzer results from single myofiber sequence ready ATAC-Seq libraries. [file elife-72792-data1.zip › figure 1- figure supplement 1-source data 1.png]

MuSC1  
MuSC2  
MuSC3

Uninjured Myofiber

MuSC1  
MuSC2  
MuSC3

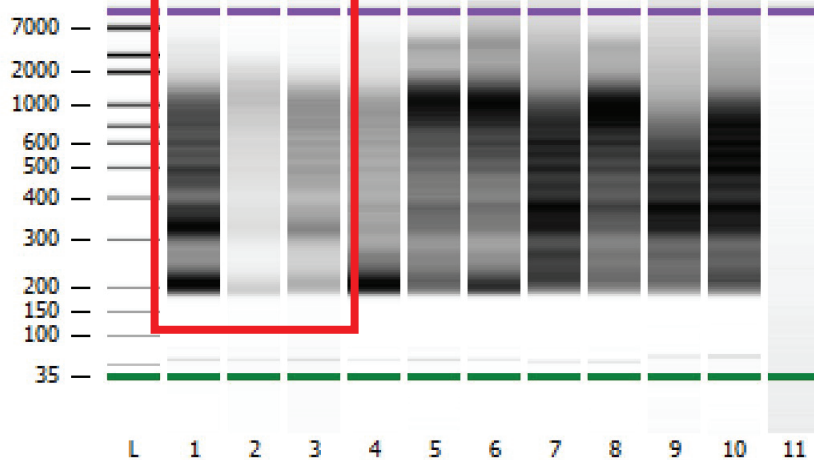

Supplement: Source data 1. — (A) Unlabeled agarose gel (1.25%) of MuSC ATAC-Seq sequence ready libraries. (B) Unlabeled agarose gel (1.25%) of uninjured myofiber ATAC-Seq sequence ready library. (C) Labeled agarose gel (1.25%) image of MuSC and uninjured myofiber ATAC-Seq sequence ready libraries. (D) Raw file of bioanalyzer results from single myofiber sequence ready ATAC-Seq libraries. [file elife-72792-data1.zip › Figure 1 figure supplement 1 labelled images source data 1.pdf]

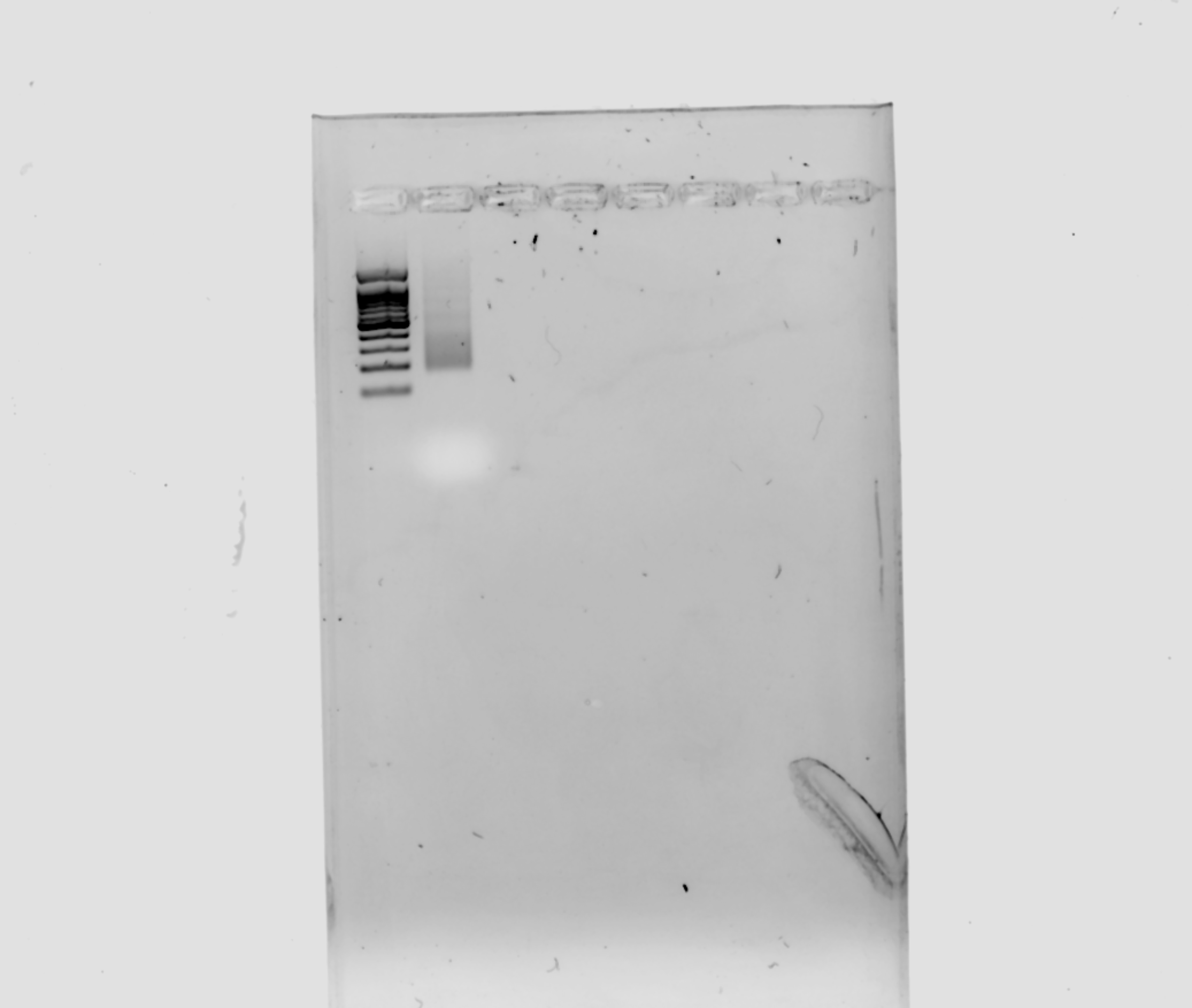

Supplement: Source data 1. — (A) Unlabeled agarose gel (1.25%) of MuSC ATAC-Seq sequence ready libraries. (B) Unlabeled agarose gel (1.25%) of uninjured myofiber ATAC-Seq sequence ready library. (C) Labeled agarose gel (1.25%) image of MuSC and uninjured myofiber ATAC-Seq sequence ready libraries. (D) Raw file of bioanalyzer results from single myofiber sequence ready ATAC-Seq libraries. [file elife-72792-data1.zip › figure 1-figure supplement 1-source data 2.png]
